# Reduced Renshaw Recurrent Inhibition after Neonatal Sciatic Nerve Crush in Rats

**DOI:** 10.1155/2014/786985

**Published:** 2014-03-23

**Authors:** Liang Shu, Jingjing Su, Lingyan Jing, Ying Huang, Yu Di, Lichao Peng, Jianren Liu

**Affiliations:** ^1^The Department of Neurology, Shanghai Ninth People's Hospital Affiliated Shanghai Jiao Tong University School of Medicine, Shanghai 200011, China; ^2^The Department of Anaesthesia, The Fifth People's Hospital of Shanghai, Fudan University, Shanghai 200240, China

## Abstract

Renshaw recurrent inhibition (RI) plays an important gated role in spinal motion circuit. Peripheral nerve injury is a common disease in clinic. Our current research was designed to investigate the change of the recurrent inhibitory function in the spinal cord after the peripheral nerve crush injury in neonatal rat. Sciatic nerve crush was performed on 5-day-old rat puppies and the recurrent inhibition between lateral gastrocnemius-soleus (LG-S) and medial gastrocnemius (MG) motor pools was assessed by conditioning monosynaptic reflexes (MSR) elicited from the sectioned dorsal roots and recorded either from the LG-S and MG nerves by antidromic stimulation of the synergist muscle nerve. Our results demonstrated that the MSR recorded from both LG-S or MG nerves had larger amplitude and longer latency after neonatal sciatic nerve crush. The RI in both LG-S and MG motoneuron pools was significantly reduced to virtual loss (15–20% of the normal RI size) even after a long recovery period upto 30 weeks after nerve crush. Further, the degree of the RI reduction after tibial nerve crush was much less than that after sciatic nerve crush indicatig that the neuron-muscle disconnection time is vital to the recovery of the spinal neuronal circuit function during reinnervation. In addition, sciatic nerve crush injury did not cause any spinal motor neuron loss but severally damaged peripheral muscle structure and function. In conclusion, our results suggest that peripheral nerve injury during neonatal early development period would cause a more sever spinal cord inhibitory circuit damage, particularly to the Renshaw recurrent inhibition pathway, which might be the target of neuroregeneration therapy.

## 1. Introduction

Recurrent inhibition (RI) is a basic type of neuronal circuit throughout the central nervous system. In the spinal cord, Renshaw cell is the only interneuron in the spinal ventral horn that receives afferents directly from motoneurons and mediates recurrent inhibition back to the motoneuron themselves, through the coreleased inhibitory neurotransmitters of glycine and GABA [1–7]. Renshaw cells and motoneurons thus form a recurrent inhibitory circuit that controls motor output. Individual Renshaw cell receives inputs from particular motor pools and spreads its inhibitory output to the same motoneurons, their synergists (i.e., motor pools exerting a similar action on the same joint), and sometimes selo ected motor pools across joints [2]. The RI is one of the important regulatory mechanisms synchronizing all muscles contract action [1–3], thus playing an important gated role in spinal motion loop [2, 8]. Renshaw inhibition dysfunction or even Renshaw cell loss has been attributed to the cause of the motoneuron degeneration in pathological conditions such as amyotrophic lateral sclerosis [4, 9, 10]. Sciatic nerve injury is a common disease of peripheral nerve in clinic with complex pathophysiological mechanism. Following nerve injury, the regenerated muscle and motoneuron were functionally disturbed, such as denervated amyotrophy and myoceptor degeneration [11]. These factors restricted functional recovery of the injured neurons. The findings that motoneuron was able to resubject muscle by regeneration and finally full or nearly full recovery in adult animals with sciatic nerve injury [12], but with reduced excitatory postsynaptic potentials, indicated the plasticity of motoneuron central synapses after transiently losing a link between motoneuron and its controlled muscle [13–15]. The RI might act as a variable gain regulator at the motoneuronal level rather than modifying the pattern of motor activity. Studies on the cats primarily demonstrated that axon function degeneration makes injured motoneuron axon collateral die out [16, 17] and adjacent neuron transiently decreased after a part of peripheral nerve axon regenerated which had been cut off then back to normal upon nerve regeneration [18]. Therefore, we presumed that spinal motor reflex is enhanced due to weak motoneuron RI because of axon abscission [19].

Our own previous study indeed demonstrated that after sciatic nerve crush at adult, the RI after the subsequent nerve regeneration was likely permanent impaired, despite the fact that the injured spinal motoneurons and the related hindlimb muscles were fully recovered [20]. However, there were no reports, so far, about the influence on spinal RI by sciatic nerve injury during early neonatal period. Our current research further investigated the relationship between RI changes and the motoneuron functional self-regulation in adult rats after transient neonatal sciatic nerve crush injury, which had a significant meaning to identify mechanism of nerve regeneration.

## 2. Materials and Methods

### 2.1. Animals

Wistar rats were maintained on an ad libitum feeding schedule and kept on a 12 hr on/off light cycle at 22 ± 1°C. All animal experiments were approved by the local committee of Laboratory Animals of Jiao Tong University School of Medicine and carried out in accordance with Chinese National Science Foundation animal research regulation. At the end of the experiments, the rats were euthanized by anesthetic overdose.

### 2.2. Peripheral Nerve Crush Injury in 5-Day-Old Rats

The peripheral nerve crush model is the preferred model for mimicking the pathophysiology that occurs most commonly in humans [21]. The basic principle of the nerve crush model is to use an injury device to deliver a force that can be adjusted to control the contusion severity. Thus, in 5-day-old baby rats, under ice cold anesthesia, the left hindlimb was exposed in the middle of the thigh and the sciatic nerve was then crushed with fine watchmaker forceps for five seconds in the popliteal fossa. In some preparations, nerve crush was carried out on tibial (Tib) nerve where the nerve crush site was below the branching point of the peroneal and tibial nerves and above the branching point of gastrocnemius-soleus nerves; thus, the crush site on the tibial nerve was about 3 mm away from the point where nerve enters into the gastrocnemius-soleus muscles (Figure 5(a)). It was (about 3 mm) closer to the muscles than that crushed on sciatic nerve (about 10 mm), which led to the result that the disconnected muscle after tibial nerve crush could be reinnervated in shorter time than that in sciatic nerve crush. After the crush, care was taken to preserve the epineurium, to facilitate regeneration of the nerves along their endoneural sheaths. This was done by visual control under a dissecting microscope. The skin was then sutured. When the animals recovered from the anesthetic, the baby rats were returned to their mother. Following nerve crush, rats were allowed to recover for at least 7 weeks and reinnervation of muscles was allowed to proceed unhindered.

Sham or injured rats were then randomly divided into groups as follows: Sham operated 5-day-old rats (Sham), 5-day-old sciatic nerve crush rats with 7-week reinnervation (5SC7), 5-day-old sciatic nerve crush rats with 30-week reinnervation (5SC30), and 5-day-old tibial nerve crush rats with 14-week reinnervation (5TC14).

### 2.3. Electrophysiological Experiment in Reinnervated Adult Rats

Rats recovered from the nerve crush at 5 days old at 7, 14, or 30 weeks were reanesthetized with *α*-chlorose (400 mg/kg, i.p.). The absence of a withdrawal reflex was used to judge the anesthesia level during the whole experimental period, and additional anesthetic (*α*-chlorose, 100 mg/kg, i.p.) was administered when necessary. During the experiment, body temperature was maintained at 37.0 ± 0.5°C with a heating blanket (Harvard Apparatus Limited, Edenbridge, UK) similar to previously reported [22].

**Figure 1:**
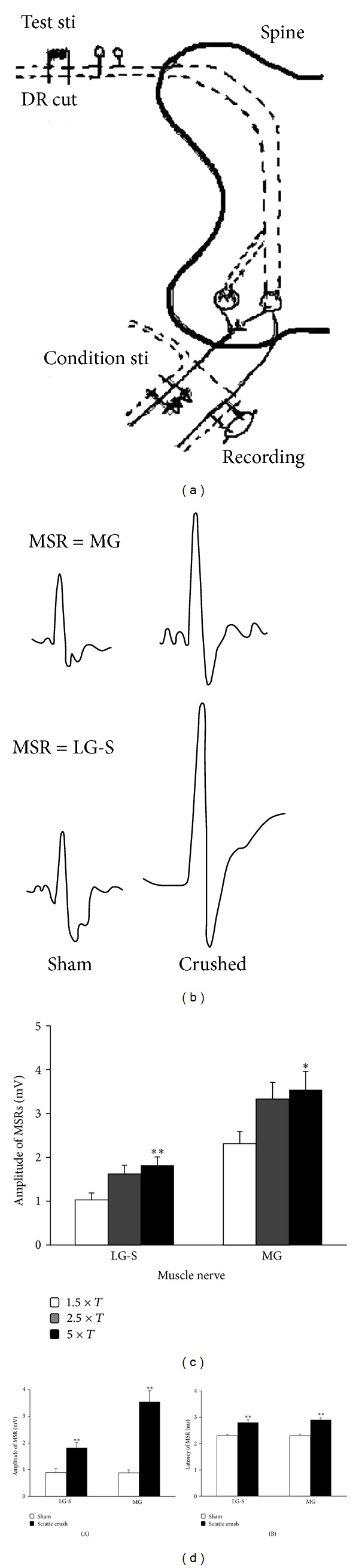
Characterization of the monosynaptic reflexes (MSR) in sham and sciatic nerve crush rats. (a) Illustration to show the test, condition stimulation, and recording arrangement during the experiment; (b) examples of the recorded MSR in sham and sciatic nerve crush rat; (c) Bar histogram showing the relationship between monosynaptic reflexes amplitude and the stimulus intensity; (d-e) bar histogram showing the monosynaptic reflexes amplitude (b) and latency (d) recorded from LG-S and MG nerves in sham (open) and sciatic nerve crushed adult rats. (**P < 0.01 in comparison with the sham control rats.)

Left ventral aspect hind leg was operated and the lateral gastrocnemius-soleus (LG-S) nerve and medial gastrocnemius (MG) nerve were separated from sciatic nerve and its branch then cut off the remote end in order to recording or stimulated. The back closed side of the fifth lumbar (L5) dorsal nerve root was cut off then stimulated for evoking monosynaptic reflexes (MSRs). Rat was then moved to stereotaxic instrument. The exposed vertebra and leg tissue and skin were fixed on both sides of stereotaxic frame to form a cube-pool filled with 37°C paraffin. Three pairs of silver bipolar electrode (0.5 mm silver, WPI) was used to either stimulate or record. Stimulation testing electrode (E1): it is put into spinal petrolin cube-pool and fixed with dissociated point of L5 dorsal nerve root to activate MSRs; MSRs recording electrode (E2): it is put into hind leg petrolin cube-pool and fixed with incomplete branch of near point of MG nerve or G-S nerve to record MSRs; adverse conditions stimulation electrode (E3): it is put into hind leg petrolin cube-pool and fixed with incomplete branch of near point of MG nerve or G-S nerve to produce reverse impulse (see Figure 1(a)).

### 2.4. Electrophysiology Data Acquisition and Analysis

Dorsal root of L5 was stimulated at a frequency of 1 Hz and intensity of 2–5 V to evoke MSRs, and the stimulus strength related to input/output relation was first studied to explore the threshold for MSR generation and the maximal response of the MSR (Figure 1(b)). The maximal MSR response was later used for all the experiments with the stimulus strength at 5x-threshold. Recorded MSRs were amplified (×1000) (Neurology System, Digitimer, UK) and input to a computer via CED1401 and processed by Spike2 (Cambridge Electronics, UK). Condition stimulus was delivered 0–50 ms before testing stimulus, and the interval was set at 0, 2, 3, 4, 5, 8, 15, 30, 40, and 50 ms. RI was expressed as the percentage reduction of the MSR amplitude.

### 2.5. Horseradish Peroxidase (HRP) Retrograde Marked G-S Motoneuron Pool

In some of the 5SC7 group animals, the exposed left G-S muscle was injected with 20% (1 uL) HRP into both inner and outer sides of the muscle. 48 h after HRP injection, animals were perfused and postfixed for 4 hours, before the spinal section of L4–L6 was dissected out. Frozen section was cut for immunohistochemistry by using Hanker-Yates solution (Hanker-Yates reagent 150 mg, dimethyl arsenic acid salt buffer 100 mL, 1% H_2_O_2_ 1 mL). The spinal sections were then stained for 15–25 min with gallocyanin (heating 10 g chrome alum dissolved into 100 mL ultrapure water, added 0.3 g cyanin) and mounted on coverslips and dried 24 h at room temperature.

### 2.6. Muscular Tissue Staining

At the end of each acute experiment (7 weeks after the initial nerve crush on day 5 of the age), the gastrocnemius and soleus muscles on both sides were dissected out and weighted separately. Gastrocnemius muscles were then quickly stored in liquid nitrogen for later SDH staining. Frozen sections (10 *μ*m) were cut, mounted on coverslips, and then dried at room temperature. After stained with hematoxylin, sections were stained again with succinic dehydrogenase (SDH) by Nicholas method (0.1 mol/L phosphate buffer 32.8 mL, 1 mol/L succinic acid sodium solution 2 mL, 15 mmol/L nitrogen blue four thiazole solution 4 mL, 0.1 mol/L KCN 0.4 mL and 10 mmol/L phenazine-N-metilsulfate solution 0.8 mL).

### 2.7. Data Analyses and Statistics

Data were presented as mean ± SEM. Student's *t*-test and One-way ANOVA with post hoc Newman-Keuls test were used to detect statistical differences. *P* < 0.05 was considered as statistically significant.

## 3. Results

### 3.1. Effect of Sciatic Nerve Crush in 5-Day-Old Rats on the MSR in Adulthood after Reinnervation

MSRs were recorded in the two branches of the sciatic nerve, LG-S and MG nerve, respectively, by stimulating the peripheral sectioned spinal root L5 (Figures 1(a) and 5(a)). The evoked MSR was stimulus strength dependent; the higher the stimulation current is, the larger the MSR responses are (Figure 1(c)). The maximal response of the MSR was chosen for late experiments. Seven weeks after sciatic nerve crush performed at day 5 after birth, the evoked MSR in crushed animals showed longer latency and larger responses. The averaged amplitude for recording from LG-S was 0.89 ± 0.15 mV (*n* = 12) in sham control rats and 1.82 ± 0.19 mV (*n* = 10) in sciatic nerve crushed rats (*P* < 0.01) and the averaged amplitude for recording from MG was 0.87 ± 0.11 mV (*n* = 12) in sham control rats and 3.54 ± 0.42 mV (*n* = 10) in sciatic nerve crushed rats (*P* < 0.01) (Figure 1(d)(A)). The mean latency of MSRs of LG-S was 2.30 ± 0.05 ms (*n* = 12) in sham control rats and 2.80 ± 0.10 ms (*n* = 10) in sciatic nerve crushed rats (*P* < 0.01) and the mean latency of MSRs of MG was 2.30  ±  0.06 ms (*n* = 12) in sham control rats and 2.90  ±  0.08 ms (*n* = 10) in sciatic nerve crushed rats (*P* < 0.01) (Figure 1(d)(B)).

### 3.2. Effect of Sciatic Nerve Crush at 5-Day-Old Rats on the RI of MSRs after 7-Week Recovery

**Figure 2:**
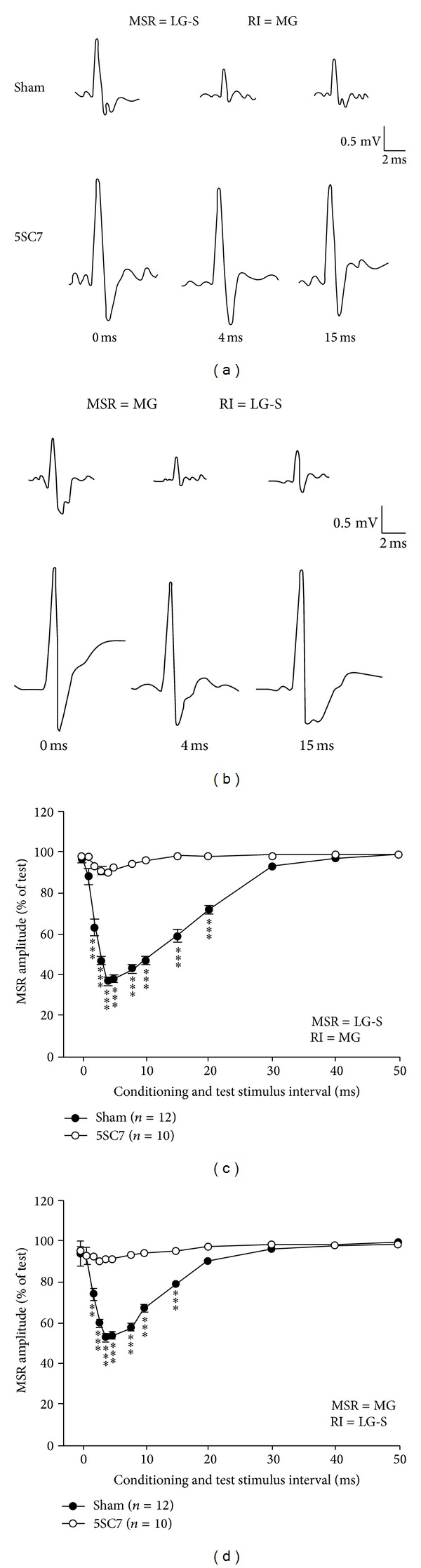
Recurrent inhibition in 5-day-old sciatic nerve crush rats—(1) 7 weeks recovery time. Recurrent inhibitory curves illustrate the amount (mean ± SEM) of recurrent inhibition (RI) of monosynaptic reflexes (MSR) in the 5-day-old sciatic nerve crush rats 7 weeks after nerve crush. The RI/MSR combinations tested were MG/LG-S (a, c) and LG-S/MG (b, d). (a-b) Raw traces of the recorded MSR from either LG-S (a) or MG (b) in different condition-test stimulus interval of 0, 4, and 15 ms from either sham control rat (upper traces) or sciatic nerve crush rat (lower traces). (c-d) recurrent inhibitory curves showing the time course of the RI in different condition-test stimulus interval. (**P < 0.01 and ***P < 0.001 in comparison with the sham control rats.)

RI was measured by applying conditioning stimulus on one sciatic nerve branch with an interstimulus interval before the test stimulus on the sectioned L5 root while recording was on the other sciatic branch.The MSR amplitude decrease indications of the RI [1]. Our results showed that, in normal condition in sham operated animals, the MSR amplitude was interstimulus interval related suppressed, with the maximal change (RI_*Max*⁡_) occurring at interstimulus interval of 4 ms (Figure 2), and the duration of the RI in this preparations was about 15–20 ms, similar to previously reported [20]. However, the RI was severely impaired by the sciatic nerve crush performed when the rats were only 5 days old after birth. Compared with the RI obtained from sham control rats in the same RI/MSR combinations, the mean values of the RI between regenerated LG-S and MG motoneurons were all significantly (*P* < 0.001, One-way ANOVA) smaller than those from the sham control group (Figures 2(c) and 2(d)). Measured at 7 weeks after the nerve crush, the maximal RI (RI_*Max*⁡_) from MG to LG-S was significantly reduced from a sham control value from 62.7  ±  3.6% (*n* = 12) to 10.1 ± 2.0% (*n* = 10) in sciatic nerve crushed rats (*P* < 0.001) and the RI_*Max*⁡_ from LG-S to MG was reduced from 47.1 ± 3.0% (*n* = 12) in sham control rats to 9.5 ± 1.8% (*n* = 10) in sciatic nerve crushed rats (*P* < 0.001) (Figures 2(c), and 2(d)). The duration of RI in this preparation was also much smaller than that in control animals. The test-conditioning stimuli interval time to get to maximal MSR depression, however, had the same value as that in sham control rats. These results indicated that when the nerve crush was applied to sciatic nerve at its early postnatal life, the RI could be depressed dramatically even when the regeneration has completed, which is different from the adult sciatic nerve crush preparation [20].

### 3.3. Effect of Sciatic Nerve Crush on 5-Day-Old Rats on the RI of the MSRs after 30-Week Recovery

**Figure 3:**
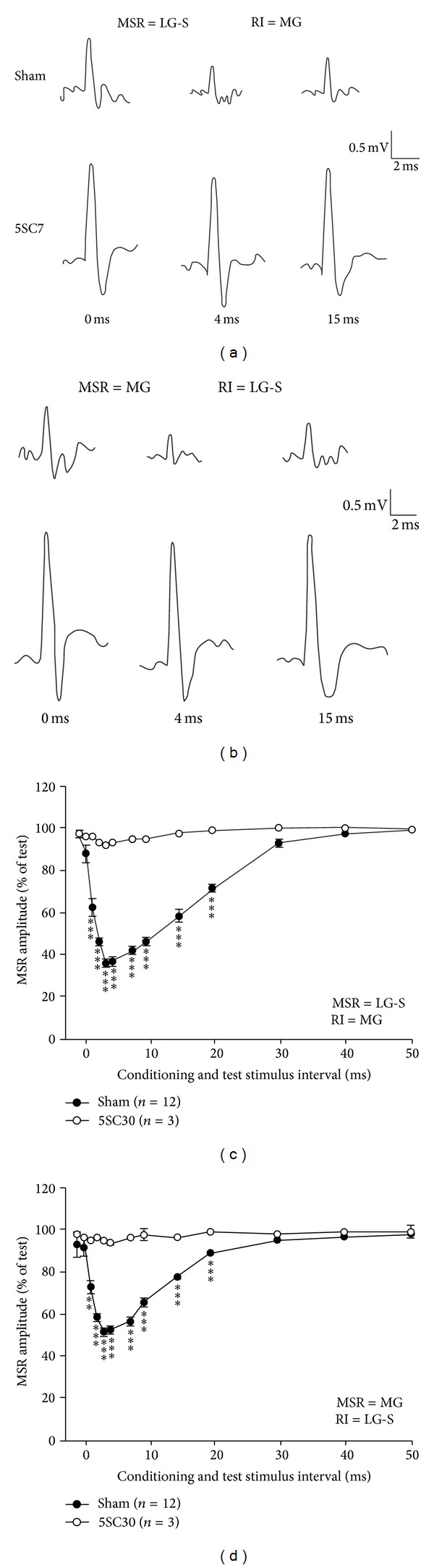
Recurrent inhibition in 5-day-old sciatic nerve crush rats—(2) 30 weeks recovery time. Recurrent inhibitory curves illustrate the amount (mean ± SEM) of recurrent inhibition (RI) of monosynaptic reflexes (MSR) in the 5-day-old sciatic nerve crush rats 30 weeks after nerve crush. The RI/MSR combinations tested were MG/LG-S (a, c) and LG-S/MG (b, d). (a-b) Raw traces of the recorded MSR from either LG-S (a) or MG (b) in different condition-test stimulus interval of 0, 4 and, 15 ms from either sham control rat (upper traces) or sciatic nerve crush rat (lower traces). (c-d) Recurrent inhibitory curves showing the time course of the RI in different condition-test stimulus interval. (**P < 0.01 and ***P < 0.001 in comparison with the sham control rats.)

**Figure 4:**
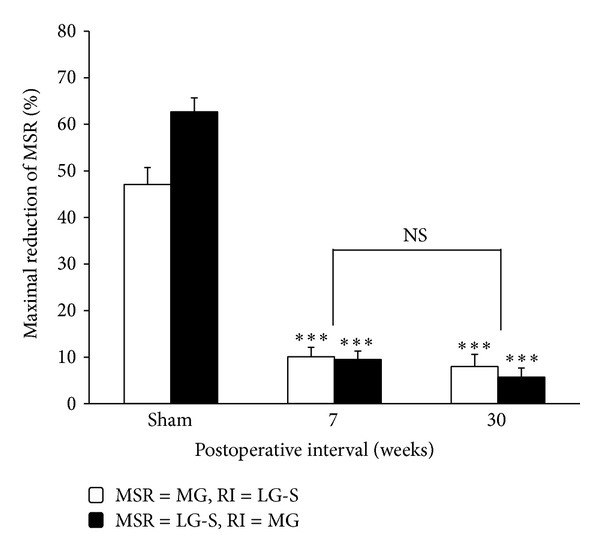
Recovery time course of the recurrent inhibition after sciatic nerve crush at age of 5 days. Bar diagram showing the recovery time course of the maximal recurrent inhibition, expressed as MSR amplitude reduction, 7 and 30 weeks after sciatic nerve crush at age of 5 days. The RI/MSR combinations tested were MG/LG-S and LG-S/MG. ***P < 0.001, two-tailed student's *t*-test.

A group of rats with sciatic nerve crush at age of 5 days were tested to check the RI change between MG and LG-S motoneuron pools 30 weeks after the nerve crush. In this preparation, a successive reduction in the amount of RI has been observed in which RI from MG to LG-S and from LG-S to MG was only capable of reducing the amplitude of MSRs to about 92.0 ± 2.6% (*n* = 3) and 94.3 ± 2.0% (*n* = 3) of the size of unconditioned MSR (Figure 3). In other words, the maximal RIs were only 8.0 ± 2.6% and 5.7 ± 2.0%, which were 12.8% (MG/LG-S) and 12.0% (LG-S/MG) of the corresponding value of the RI in sham control rats, respectively (Figure 4). The amounts of RI from this preparation were all significantly smaller than those from sham control rats (*P* < 0.001, One-way ANOVA).

In addition, comparing the group data of 7-week and 30-week postoperative nerve crush rats, the RI was not different statistically (*P* > 0.5 in MG/LG-S, *P* > 0.1 in LG-S/MG, *t*-test) (Figure 4). These results indicated that the depressive effect of the nerve injury on the spinals RI could not recover following regeneration or, in other words, this impairment seems permanent.

### 3.4. Effect of Tibial Nerve Crush at 5-Day-Old Rats on the RI of MSRs after 14-Week Recovery

**Figure 5:**
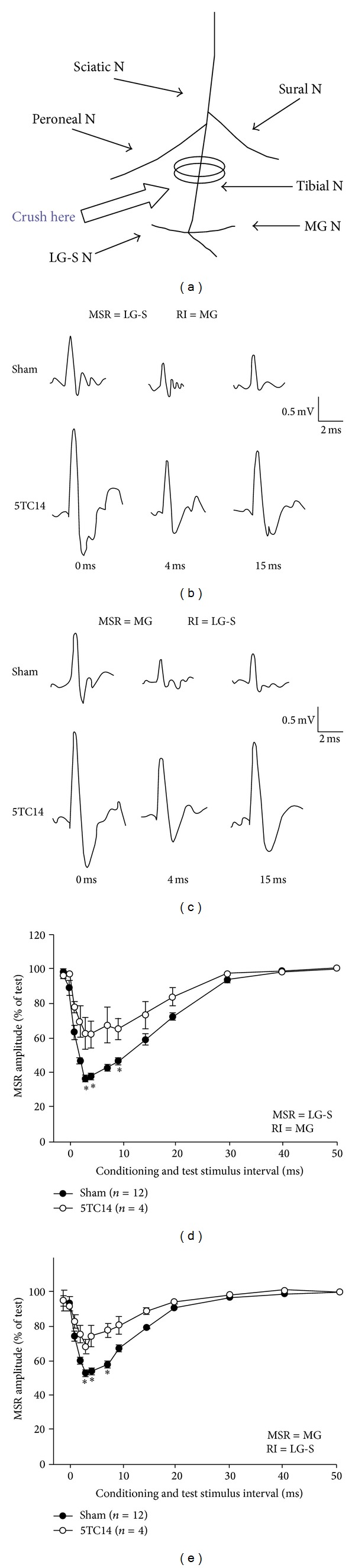
Recurrent inhibition in 5-day-old tibial nerve crush rats. (a) Illustration of the tibial nerve crush site and the relation to the recording and testing nerves. (b-c) Raw traces of the recorded MSR from either LG-S (a) or MG (b) in different condition-test stimulus interval of 0, 4, and 15 ms from either sham control rat (upper traces) or sciatic nerve crush rat (lower traces). (d-e) Recurrent inhibitory curves illustrate the amount (mean ± SEM) of recurrent inhibition (RI) of monosynaptic reflexes (MSR) in different condition-test stimulus interval. **P < 0.01 and ***P < 0.001 in comparison with the sham control rats.

In another group of the rats, tibial nerve, instead of the sciatic nerve, was crush injured at age of 5 days and the RI was examined 14 weeks later. The RIs of MSRs between regenerated MG and LG-S motoneurons 14 weeks after tibial nerve crush were all significantly reduced (*P* < 0.05, One-way ANOVA) (Figure 5(b)). The maximal RI reduction occurring in either combination was at 4 ms of interstimulus interval (Figures 5(d) and 5(e)). The RI from MG motoneurons to LG-S motoneurons maximally reduced the amplitude of the MSRs to 62.0  ±  9.2% (*n* = 4) of the size of the unconditioned reflexes, which is indication of a RI_*Max*⁡_ value of 38.0 ± 9.2% (Figure 5(d)). The corresponding value of the MSR reduction from LG-S to MG was 68.4  ±  5.3% (*n* = 4) and a RI_*Max*⁡_ of 31.6 ± 5.3% (Figure 5(e)). Comparing these results in neonatal tibial nerve crush rats with those from the sham control rats (Figures 5(d) and 5(e)), the RI_*Max*⁡_ was significantly reduced either LG-S to MG or MG to LG-S combinations (*P* < 0.05, *t*-test) (Figures 5(d) and 5(e)).

### 3.5. Effect of Sciatic Nerve Crush at 5-Day-Old Rats on the Spinal Motoneurons and the Hindlimb Muscles after 7-Week Recovery

**Figure 6:**
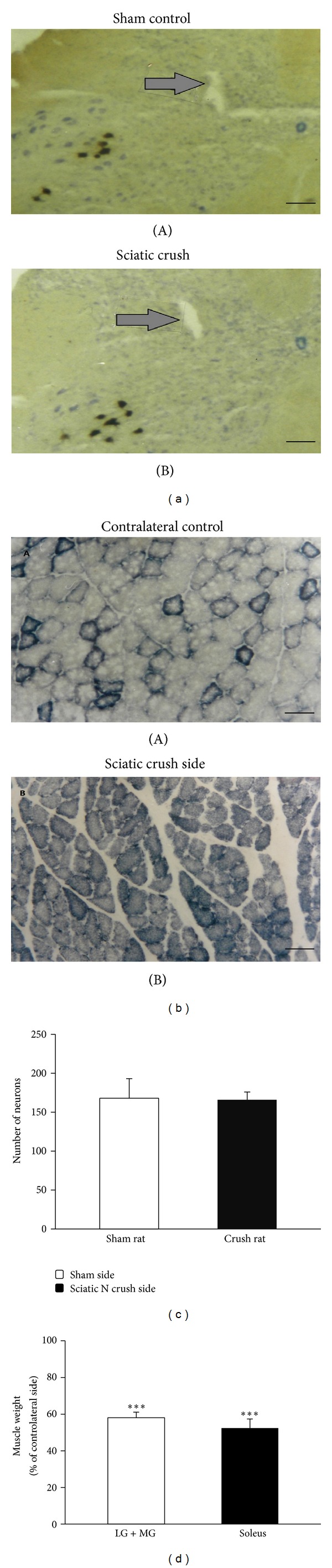
Spinal motoneuron and peripheral muscle change 7 weeks after the 5-day-old sciatic nerve crush. (a) Photograph taken under low magnification (4x) showing nerve crush side of the spinal cord with the HRP labeled cells is located on dorsolateral part of the anterior horn. The hole pointed by the arrow indicates the HRP injected side. (b) Examples of cross-sections from reinnervated (B) and contralateral control (A) gastrocnemius muscles from a 5-day-old sciatic nerve crush rat stained for SDH 7 weeks after nerve crush. (c-d) Bar diagrams showing the motoneuron number of the gastrocnemius pool (c) and gastrocnemius and soleus muscle weights (d) in 5-day-old sciatic nerve crush rats 7 weeks after reinnervation. The scale bar in ((a)(A) and (a)(B)) is 500 *μ*m and in ((b)(A) and (b)(B)) is 80 *μ*m. ***P < 0.001 in comparison with the sham control.

Our previous results indicate that, after sciatic nerve crush in adult, either the spinal motorneurons or the relative muscles were fully recovered from the initial insult after at least 6-week reinnervation [20]. Here we further examined whether sciatic nerve crush performed during early development at age of 5 days would affect the number of the spinal motorneurons and the property of the muscles after reinnervation. Our results showed that, 7 weeks after sciatic nerve crush performed at 5-day-old rats, the number of the spinal motorneurons located in the ventral side of the spinal cord was not different between the sham control group and the nerve crushed group (Figure 6(a)). There was similar number of the spinal G-S neuron among crushed group (166 ± 10, *n* = 4) and the sham control group (174 ± 12, *n* = 4) (*P* > 0.05) (Figure 6(c)). Although there was no motoneurone loss after nerve crush in 5-day-old rats, in contrast, compared to the uninjured side, the muscle weight for both gastrocnemius-soleus and tibial muscles was significantly reduced 7 weeks after sciatic nerve crush at age of 5 days (*P* < 0.05, Figure 6(d)). The gastrocllemius muscles had the weight at 58.0 ± 3.1% (*n* = 8) of the contralateral control value, and the soleus muscles were 52.4  ±  5.0% (*n* = 8) of the control value. Loss of muscle weight after peripheral nerve crush in 5-day-old sciatic nerve crush rats observed in this study is in agreement with the other results [23, 24]. In addition, the muscles, which were stained with succinic dehydrogenase (SDH) to show its oxidative capacity, showed that the denervated/regenerated muscle of gastrocnelnius muscle virtually stained darkly, in comparison with control muscle having mixture of fibers which have either a low (pale staining) or high (dark staining) oxidative capacity (Figure 6(b)). The result of muscle fiber type change is in good agreement with previous reports [23–25].

## 4. Discussion

The current research is the first to report the change of the Renshaw recurrent inhibition (RI) of spinal MSRs in rats after reinnervation following the sciatic nerve crush injury during neonatal period. The following results were obtained. (1) In 5-day-old sciatic nerve crush preparation, the monosynaptic reflexes evoked from the regenerated motoneurons not only had a longer latency but also had larger amplitude. (2) The amounts of RI between regenerated motor pools were significantly suppressed, and they were virtually lost compared with the normal RI size (15–20% of the normal RI value) even after a long recovery period (10–15% of the sham control at up to 30 weeks after nerve crush). (3) In contrast to the huge reduction in sciatic nerve crush preparation, the amounts of RI following tibial nerve crush, which was more close to their target muscles, at age of 5 days were only reduced to about 60–70% of the normal RI size. This may suggest that shortening the disconnection time between motoneurons and their target muscles would lead to less impairment in the RI pathway after nerve crush. (4) Despite being without motoneuron loss, the muscles innervated by those injured nerves were changed particularly with changed composition of the muscle fibers.

In the 1940s Renshaw discovered RI of motoneuron axon collateral inhibited spinal MSR induced by activating a group of interneurons located in the spinal motoneuron area via reverse stimulation of the axon of the motoneurons [1], thus the interneuron named Renshaw cell and the inhibition named Renshaw recurrent inhibition [2, 8]. It was reported that the Renshaw cell to motoneuron ratio is estimated to be 1 : 5 [26]. The findings from current study showing that the MSR latency was prolonged after nerve crush may attribute to decreased rate of axon transmission. The mechanism of decreased RI of neuron axon because of injury may be long term or permanent which agrees with the result of peripheral nervous regeneration after cutoff. Havton and Kellerth [27] and Kellerth et al. [28] found that motoneuron axon collateral disappeared after never injury associated with the change of RI in cats, which indicated the possibility of less axon collaterals leading to subdued RI intensity. Whether or not the axon collaterals die out in rats after sciatic nerve crush was not studied in current experiment, and further studies are certainly needed. Moreover, the lower activity of Renshaw cells was thought to relate to the decreased RI, especially in the early period of injury. Although it is unclear how Renshaw cell changes after sciatic nerve injury, Sanna and her colleagues [29] found that activity of Renshaw cells weakened upon sciatic nerve crush 1 week after injury that was likely to be a result of disappearance of axon collaterals and abnormal function of motoneurons.

Monosynaptic reflexes generated from adult injury and regenerated motoneurons and recorded in a peripheral nerve crush rat had a similar strength as in normal [20]. In contrast, monosynaptic reflexes recorded from regenerated motoneurons in 5-day-old sciatic nerve crush rats are much different from those in sham control rats not only by their latency, but also by their amplitude. However, the latencies of MSRs in regenerated motoneurons (either from adult or 5-day-old sciatic nerve crush rats) were significantly longer than those in sham control rats. This may be due to the observed reduction of axon conduction velocity in injured motoneurons [30–32]. The largely increased amplitude of MSRs seen in 5-day-old sciatic nerve crush rats was not seen in adult crush rats. This MSR amplitude increase is in agreement with the early report that after temporary loss of contact with the target in young animals, the surviving motoneurons became more active than normal; however, in animals which had their nerve crushed as adults the motoneuron activity was as normal [33]. It is possible that nerve injury in neonatal animals disrupted the normal developmental process and prevented the usual elimination of synapse from the surface of the motoneurones, which would result in enhancement of the monosynaptic EPSPs [14, 33, 34]. The enhanced monosynaptic EPSPs evoked from crushed nerve were reported only in a period of 8 to 12 weeks after nerve crush [34, 35]. Since the sciatic nerve crush carried out on 5-day-old rats in this study led to both deafferentation and denervation of the sciatic motor pool, albeit briefly, these changes of motoneuron synaptic input may result in the enhancement of MSRs.

Compared to our previous study on the adult sciatic nerve crush rats [28], it is clear that peripheral nerve injury in young animals affects the RI pathway more than that in adult. This result may relate to the findings in muscles and motoneurones [23, 24, 33, 36–40]. For example, immature motoneurones are more likely to die as a result of axonal damage than mature ones [23, 36] and even the surviving motoneurones are unable to recover the original size of their peripheral field [41]. Adult mammalian muscles recover virtually completely from nerve injury if the reinnervation is allowed to proceed unhindered [20, 36, 37]. However, the reinnervated muscles in neonatal nerve injury animals are affected to a much greater extent than after similar injury in adult animals, seen in both current neonatal nerve crush study and our previous adult sciatic nerve crush study [20]. The effects include gross loss of weight, loss of muscle tension, and muscle fiber grouping [23–25, 36, 37]. The reason why the nerve injury applied in adult and young animals induces such a different impairment to motor units has been explained as a consequence of the development of the muscle fibers being arrested while the motoneurons continue to develop after nerve injury [36]. Upon reinnervation the still immature muscle fibers may not be able to match the functional demands imposed upon them by the now mature nervous system. In the RI pathway in spinal cord, it has been found that after nerve injury the Renshaw cells in adult rat appear to be inactive during reinnervation [29] and motor axon collaterals are eliminated [16, 17]. There is also evidence showing that postnatal elimination of a large number of terminal arborization and synaptic buttons of recurrent motor axon collaterals occur during the first two weeks of postnatal life [42, 43]. This elimination of terminal axon collaterals appears to be coincident with the elimination of polyneuronal innervation which is known to occur at the neuromuscular junction [44]. That may be the factor, that during the period of inactivity of Renshaw cells following the neonatal sciatic nerve crush, the elimination of axon collaterals process underwent. Without interaction with the target (Renshaw cells), more axon collaterals of the motoneurons may be eliminated than those in the normal developmental process, since the activity of the target plays a very important role in the development of motor units. In addition, at the Renshaw cell level, after Renshaw cells restored the ability to respond synaptic input, which in adult is about 6–8 weeks after nerve injury, the postnatal recurrent axon collaterals elimination process has already finished [42, 43] and the immature Renshaw cells thus could not undergo the developing process to match their mature function. If the denervation time is long enough, it may cause the permanent change in Renshaw cell firing properties that never recover to match the mature state. In contrast, in adult animals, both motoneurons and Renshaw interneurons are mature type at the time of nerve injury and after recovery from temporary “arrest,” Renshaw cells recover to active as normal [29]. Thus, the large reduction of recurrent inhibition seen in this study in 5-day-old nerve crush rats may be accounted for by (1) the elimination of recurrent axon collaterals, (2) loss of synaptic contact with regenerated motoneurons by Renshaw cells as those in adult nerve crush rats, and (3) the abnormal excitability of Renshaw cells themselves.

In addition, in this study the length of time during which muscles were separated from their neurons was varied using three different nerve crush sites either on the sciatic nerve or moving along the nerve to the tibial branch in 5-day-old rats. Results showed that shortening the period of denervation improved recovery of the recurrent inhibitory effect. Taken together these experiments show that the degree of permanent impairment of recurrent inhibitory pathway following temporary denervation during the neonatal period is related to the length of time during which the motoneurons and muscles are disconnected. The longer the period of separation is, the more severe the impairment is. These results are supported by the other findings. Lowrie et al. [24] reported that crushing of the peroneal nerves at 3 mm away from the EDL muscle at age of 5 days was followed by a better recovery in EDL muscle than that crushing site at 9 mm away from the muscle. Brown et al. [45] demonstrated a similar result in soleus muscle in 2-day-old rat in which soleus muscle was nearly in complete recovery after a crush on soleus nerve at its point of entry into the muscle. Thus, it could be concluded that nerve crush inducing RI impairment is likely related to the time length of disruption of the motoneuron-muscle interaction.

## 5. Conclusion

The result presented in this work from the neonatal sciatic nerve crush rats, and adding our previous findings from the adult sciatic nerve crush rats, indicates that regenerated motoneurons in general showed reduced RI with the most dramatic effect being on motoneurons injured in early postnatal life and the decreased RI had a special significance in making up motoneuron's function enhance for the alteration of the muscle power.
